# Ependymal Cilia: Physiology and Role in Hydrocephalus

**DOI:** 10.3389/fnmol.2022.927479

**Published:** 2022-07-12

**Authors:** Weiye Ji, Zhi Tang, Yibing Chen, Chuansen Wang, Changwu Tan, Junbo Liao, Lei Tong, Gelei Xiao

**Affiliations:** ^1^Department of Neurosurgery, Hunan Cancer Hospital and the Affiliated Cancer Hospital of Xiangya School of Medicine, Central South University, Changsha, China; ^2^Department of Neurosurgery, Xiangya Hospital, Central South University, Changsha, China; ^3^Diagnosis and Treatment Center for Hydrocephalus, Xiangya Hospital, Central South University, Changsha, China; ^4^National Clinical Research Center for Geriatric Disorders, Xiangya Hospital, Central South University, Changsha, China

**Keywords:** ependymal cilia, hydrocephalus, cerebrospinal fluid, pathogenesis, treatment

## Abstract

Cerebrospinal fluid (CSF), a colorless liquid that generally circulates from the lateral ventricles to the third and fourth ventricles, provides essential nutrients for brain homeostasis and growth factors during development. As evidenced by an increasing corpus of research, CSF serves a range of important functions. While it is considered that decreased CSF flow is associated to the development of hydrocephalus, it has recently been postulated that motile cilia, which line the apical surfaces of ependymal cells (ECs), play a role in stimulating CSF circulation by cilia beating. Ependymal cilia protrude from ECs, and their synchronous pulsing transports CSF from the lateral ventricle to the third and fourth ventricles, and then to the subarachnoid cavity for absorption. As a result, we postulated that malfunctioning ependymal cilia could disrupt normal CSF flow, raising the risk of hydrocephalus. This review aims to demonstrate the physiological functions of ependymal cilia, as well as how cilia immobility or disorientation causes problems. We also conclude conceivable ways of treatment of hydrocephalus currently for clinical application and provide theoretical support for regimen improvements by investigating the relationship between ependymal cilia and hydrocephalus development.

## Introduction

Hydrocephalus is a neurological disease caused by an excess of cerebrospinal fluid (CSF) in the ventricles (Filis et al., 2017). The classic definition of hydrocephalus refers to two types: communicating and non-communicating (also known as obstructive) (Shim et al., 2019; Zhang J. X. et al., 2020). Excess fluid is present in communicating hydrocephalus due to abnormalities in CSF secretion, motility, and/or absorption (Finn et al., 2014; Shim et al., 2019). Blockage of the CSF flow during circulation causes obstructive hydrocephalus (Højlund et al., 2018). Headache, lower-limb weakness, starting or gait instability, urine incontinence, ataxia, and progressive loss of autonomic language and physical activity are also typical symptoms (Filis et al., 2017). It could be congenital or the result of excessive CSF production, malabsorption, or obstruction due to trauma, infection, venous occlusion, tumors, intracranial hemorrhage, or motile cilia dysfunction (Shim et al., 2019).

Cerebrospinal fluid is a transparent, colorless liquid found in the ventricle and subarachnoid region. It is primarily produced by the choroid plexus in the lateral ventricle, third ventricle, and fourth ventricle, with some directionality in its flow (Berliner et al., 2019; Lloyd et al., 2020). CSF is created, absorbed, and returned to the vein on a constant basis. In the central nervous system, it functions similarly to lymph. It provides specific nutrients to brain cells, transfers metabolites from brain tissue, and maintains the acid-base balance of the central nervous system. To relieve pressure on the brain and spinal cord, cerebrospinal fluid (CSF) surrounds and supports them.

Heart and cilia are two power sources of the circulation of CSF. Cilia are highly conserved organelles that protrude from the surface of nearly every cell type and are classified into two types: motile and primary cilia (Mitchison and Valente, 2017; Zhang J. et al., 2020). Respiratory epithelial cells, oviduct cells, node cells, and ECs all have mobile cilia (Zhang J. et al., 2020). Ependymal cilia, a long process that can swing out of the free surface of ECs, are found in the central nervous system (CNS) (Dur et al., 2020) and extend into the myelocoele. Normal ependymal cilia are uniformly distributed on ECs and have a constant length. The special unique plane polarity of cilia guarantees that they swing in a stable direction during movement, promoting directed CSF circulation in the brain (Kumar et al., 2021; Yamada et al., 2021).

As a result, it goes without saying that cilia play an irreplaceable role in the development of hydrocephalus. The aberrant accumulation of CSF in the ventricle is caused by the malfunction of ependymal cilia, which obstructs the normal flow of CSF, resulting in an imbalance between CSF production and absorption (Al Omran et al., 2017). Despite the fact that the physiology and significance of ependymal cilia in hydrocephalus has aroused researchers’ curiosity, there are still some limits in this field. Thus, we’ll focus on the physiological activities of ependymal cilia in this review, as well as the mechanism of cilia immobility, or cilia disorientation, which leads to disorders. Furthermore, we attempt to decipher the process of hydrocephalus development based on factors that cause pathophysiology of motile cilia, such as alcohol abuse, degenerative neuropathology, PCD, and brain injury, and thus propose conceivable treatment options for hydrocephalus, which may provide new theoretical support for future research by examining the relationship between ependymal cilia and hydrocephalus development.

## Physiological Functions of Ependymal Cilia

Ependymal cilia, which are found in the ventricular system of the brain (Ringers et al., 2020), serve an important role in maintaining appropriate CSF flow (Chiani et al., 2019). Ependymal cilia ensure CSF flow and are necessary for CSF homeostasis and directional migration of neural cells.

### Ependymal Cilia Propelling the Circulation of Cerebrospinal Fluid

The directional flow of CSF depends directly on ependymal cilia within the ventricular system (Lee, 2013; Kumar et al., 2021). Ependymal cilia can be found on ECs all across the brain, projecting up to 20 microns from the cell (Zhang J. et al., 2020). As CSF circulation requires a powerful and accurate pressure from the exterior (Kumar et al., 2021), the synchronized beats of ependymal cilia that plays an indispensable role in the flow of CSF from the lateral ventricles to the third and fourth ventricles, and then toward the subarachnoid space for absorption (Vidovic et al., 2018). This mechanism was recently confirmed by Alexia Mahuzier’s experiment, which looked at mice with mutations in genes important for cilia development.

Ependymal cells have about 50 motile cilia per cell, which aid in directing CSF flow through the ventricular system. The rotating organization of the basal bodies (BBs) is required for the coordinated beat of ependymal cilia. Rotational polarity refers to the unidirectional arrangement of all basal feet (BF) on each EC. BF protrude unilaterally from the BB barrel, affecting CSF flow direction (Mirzadeh et al., 2010; Wallingford, 2010; Ohata et al., 2014; Ryu et al., 2021). It is clear that cilia motion is indispensable for initiating and maintaining apical actin enrichment at the apex of centriolar plaque. The apical enrichment of actin in centriolar plaques in adult ECs helps to the maintenance of the optimum number and spacing of centrioles, allowing ECs to maintain an ideal number of motile cilia and hence ensure effective CSF flow in the ventricles (Mahuzier et al., 2018).

Furthermore, the continual beating of ependymal cilia aids in CSF nutrition exchange and waste clearance (Xiong et al., 2014; Fame and Lehtinen, 2020), assisting in the maintenance of CSF homeostasis.

### Ependymal Cilia Regulating Neuroblast Migration

**FIGURE 1:**
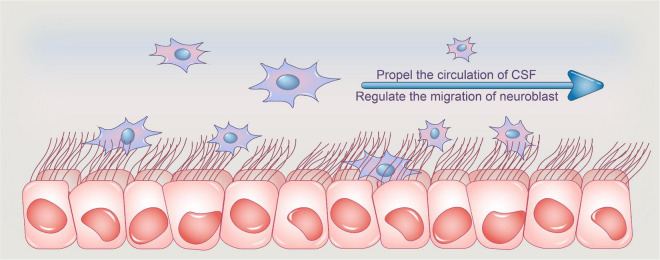
Functions of ependymal cilia. Ependymal cilia protrude from ependymal cells, and their synchronized pulsing transports CSF from the lateral ventricle to the third and fourth ventricles, where it is absorbed. Adult ependymal cells’ apical enrichment of actin in centriolar plaques aids in the maintenance of the optimal number and spacing of centrioles, allowing ependymal cells to maintain an ideal number of motile cilia and thus aid in CSF nutrition exchange and waste clearance, assisting in CSF homeostasis. Furthermore, the beating of ependymal cilia can propel CSF flow and create a concentration gradient of guiding molecules, which aids neuroblast migration orientation. CSF, cerebrospinal fluid.

Previous study has revealed that ependymal cilia in the brain ventricles direct neuroblast migration in both adult and mouse development (Marshall and Nonaka, 2006; Sawamoto et al., 2006). The beating of ependymal cilia can propel CSF flow and form a concentration gradient of the guiding molecules, facilitating the orientation of neuroblast migration (Hirschner et al., 2007). Interestingly, a recent study found that the Xenopus neuroblast also relies on ependymal cilia-driven CSF flow, owing to the ability of the polarized ependymal cilia to create a polarized flow (Dur et al., 2020; Figure 1, Functions of ependymal cilia).

## Lack of Ependymal Cilia

Ciliary abnormalities include the absence of cilia, cilia immobility, and alterations in planar polarity that shift the direction of the cilia’s beat (Wu et al., 2020). The relationship between cilia failure and gene mutations has been actively explored in humans thanks to developments in research methodologies, and genetic analysis of human hydrocephalus cases have been continuously reported. As a result, we’ve compiled a list of linked pathogenic genes discovered in hydrocephalus induced by ependymal cilia abnormalities.

Although numerous gene mutations that we have known can cause loss of ependymal cilia to varying degrees, we will review genes that cause cilia deletion according to a novel concept in this review. Cilia are derived from BBs, and the centrioles are the primary structure of the BBs. As a result, numerous dysfunctions of the centriole will interfere with the normal proliferation of ependymal cilia, resulting in cilia loss. Therefore, we’ll focus on the genes linked to ciliary loss caused by centriole dysfunctions.

FoxJ1 is a transcription factor that is required for ciliogenesis in multiciliated cells (MCCs) of the mouse (Lewis and Stracker, 2021). The expression of γ-tubulin, dynein, and kinesin motor proteins is severely lacking in FoxJ1-deficient mice, resulting in the inability of centriole to be transported to the surface of ECs, impairing the formation of ependymal cilia (Jacquet et al., 2009). Human patients with the FoxJ1 mutation have recently shown motor cilia abnormalities caused by BBs injury in MCCs (Lewis and Stracker, 2021).

Mcidas (Mci or Idas), Geminin protein, GemC1 (also known as Gmnc or Lynkeas), and FoxJ1 are transcription regulators that play a pivotal role in the formation of ECs. Both GemC1 and Mcidas have been found in studies to induce MCC differentiation in the brain. Mcidas, in combination with transcription factors such as TAp73, c-Myb, E2F4 and E2F5, increases the development and maturation of BBs according to several studies (Omiya et al., 2021). However, Mcidas-deficient mouse developed morphologically discernible MCCs in the brain, which can express early transcription factors such as p73 and FoxJ1, but cannot extend centrioles, thus resulting in the loss of ependymal cilia (Lewis and Stracker, 2021).

NHERF1/EBP50 is an adapter protein found mostly in the apical plasma membrane of human epithelial cells. NHERF1-deficient mice’s lateral ventricle, third ventricle, and fourth ventricle swelled, indicating the formation of non-obstructive hydrocephalus and it can be either mild or severe. NHERF1 expression was highest at the apical of plasma membrane (PM) of ECs, and was involved in the emergence of non-obstructive hydrocephalus through the disruption of ciliary movement of ECs, according to brain sections (Georgescu et al., 2015).

Odf2 is a gene that encodes a highly stable fibrous structure in the sperm tail and was first discovered as one of the main components of the outer dense fiber (ODF). When exons 6 and 7 of Odf2 are deleted, mice’s cilia lose a considerable amount of their basal feet. This structure is linked to the basal body triad and aids in the formation of BBs. Furthermore, it has been demonstrated by immunofluorescence that the number of cilia per cell decreased in *Odf*2^Δ*Ex*6,7/Δ*Ex*6,7^ MCCs in the ventricle (Kunimoto et al., 2012).

Cyclin O, encoded by CCNO, is a member of the cyclin family that regulates ciliogenesis and apoptosis. CCNO mutations have recently been discovered in human patients and animal models, and they can result in the entire loss or severe decrease of ependymal cilia. CCNO is required for the proper production and maintenance of deuterostomes, a cytoplasmic structure responsible for the expansion of centrioles, which attach on the plasma membrane during cilia formation to form BBs, which serve as a platform for the growth of ciliary axonemes. The CCNO mutation causes Ccno deficit, which causes deuterostomes to fail to form correctly, resulting in a decrease in the number of centrioles and the inability to grow cilia regularly, thus leading to the loss or insufficient number of ependymal cilia (Wallmeier et al., 2014; Funk et al., 2015; Núnez-Ollé et al., 2017).

NME7, also known as non-metastatic cell 7 or nucleoside diphosphate kinase 7, is a member of the NME family of proteins that modulates the nucleation activity of γ-tubulin ring complex (γTuRC) and helps to regulate the microtubule (MT) organizing center. The γTuRC is the principal MT nucleator in animal cells and plays a crucial role in MT organization. Inhibition of NME7 expression in ependymal cilia suppresses centrosome-based MT nucleation (Liu et al., 2014; Šedová et al., 2021), impairing the normal development of centrioles and therefore resulting in the loss of ependymal cilia.

Huntington protein (HTT) is a gene that regulates ciliogenesis by interacting with Huntington-associated protein 1 (HAP1) and perinuclear matter 1 protein (PCM1). The depletion of HTT in ECs caused ciliary layer alterations in mice. HTT or HAP1 deficiency caused PCM1 to scatter from centrosomes, resulting in impaired ciliogenesis *in vivo* and hydrocephalus in mice. In Huntington Disease (HD), aberrant ependymal cilia disrupt CSF flow and interfere with neuroblast migration, disrupting brain homeostasis and speeding up disease progression (Keryer et al., 2011).

## Immobility of Cilia

According to previous research, each ependymal cilium has a central pair of singlet MTs known as the central pair (CP) complex and nine parallel doublet MTs known as peripheral microtubules (PMTs), forming a so-called ‘9 + 2’ structure. Additionally, the outer component is known as the outer dynein arm (ODA), whereas the inner structure is known as the inner dynein arm (IDA). A radial spoke structure in each PMT regulates motility by connecting the CP to the dynein arms (Marshall and Nonaka, 2006; Sawamoto et al., 2006). As a result, there is little question that if the ependymal cilia are structurally abnormal, they will be unable to swing normally, eventually affecting the normal circulation of CSF.

### Altered Structure of Axonemes

Mice lacking the Jhy gene (Jhy^lacZ/lacZ^) developed dilated lateral ventricles and juvenile hydrocephalus as early as 1.5 days after birth. The ependymal cilia lining the lateral ventricles of Jhy^lacZ/lacZ^ animals are sparser, shorter, more irregular, and disoriented at higher magnification, implying that they may have no function. The ‘9 + 2’ MTs in the cilia of Jhy^lacZ/lacZ^ animals were disorganized, rather, most of the cilia of Jhy^lacZ/lacZ^ animals formed axonemes with ‘9 + 0’ or ‘8 + 2’ MTs, according to ultrastructural studies. Ciliary motility is assumed to be dependent on the CP of ‘9 + 2’ cilia, which also controls the direction of the beat. The aberrant ependymal cilia have ‘9 + 0’ confirmation, suggesting that Jhy^lacZ/lacZ^ ‘9 + 0’ cilia are nearly immotile. As a result of Jhy gene mutations, these aberrant ependymal cilia are unable to maintain sufficient CSF flow, which appears to be the cause of hydrocephalus in Jhy^lacZ/lacZ^ mice (Appelbe et al., 2013; Muniz-Talavera and Schmidt, 2017).

### Altered Structure of Dynein Arms

Dynein axonemal assembly factors (DNAAFs), which refer to a group of proteins, are involved in the preassembly of ODA complexes. The basic structures of ciliary movement are dynein arms. By hydrolyzing ATP, ODA provides the right mechanical force for ciliary beat, whereas IDA controls the ciliary beating mode. ODA complexes are preassembled in the cytoplasm before being transported to ciliated axons. DNAAF1 [also known as outer dynein arm 7 (ODA7) or leucine-rich repeat-containing protein 50 (LRRC50)], DNAAF2 [also known as PF13 or kintoun (KTU)], DNAAF3 (also known as PF22), and DNAAF4 (also known as DYX1C1) are the four types that have been discovered so far (Inaba et al., 2016). DNAAFs work with molecular chaperones to ensure that ODA subunits fold correctly, allowing ependymal cilia to move normally. The preassembly of ODA was impaired when DNAAFs mutated, making the ependymal cilia unable to swing normally (Ha et al., 2016).

Except DNAAFs, leucine-rich repeat-containing protein 6 (LRRC6) is also required for the transport of the ODA complex from the cytoplasm to the cilia. The structure of ependymal cilia remains intact in the absence of LRRC6, but they are entirely immobile. The ‘9 + 2’ structure of MTs remains normal in mice lacking LRRC6, but ODA proteins that should be carried to the ciliary axonemes remain in the cytoplasm and are not delivered to the ciliary axonemes, according to studies. The cilia in the ependymal cilia lack ODAs, which are necessary for cilia movement, resulting in cilia immobility (Inaba et al., 2016).

The inner and outer dynein arms, as previously stated, are essential components for cilia movement. The coiled-coil domain-containing protein 151 (CCDC151) gene encodes a coiled-coil protein that aids in the assembly of the ODA complex and ensures that it is appropriately linked to MTs. Since we already know that dynein’s mechanical force aids in the synchronous beating of ependymal cilia, a recent experiment confirms our previous findings by showing that the loss of CCDC151 causes complete loss of ODA in axonemes, resulting in severe ciliary dyskinesia, and most of the ependymal cilia around the ventricle were immobile (Chiani et al., 2019).

DNA Polλ, commonly known as Polβ2, is a key gene for IDA assembly and belongs to the PolX family. Aside from electron microscopy, investigations have indicated that in mice lacking DNA Polλ, the dynein arms of cilia from the EC layer are defective. Furthermore, ependymal cilia without ODAs can beat slowly, but IDAs appear to be essential. As a result, it appears that ciliary immobility seems to be blamed to defective IDAs, which are induced by DNA Polλ mutants (Kobayashi et al., 2002). However, there is still considerable disagreement. Zariwala et al. (2004) recently looked into the deletion construct further and discovered that in the mouse model, the expression of a new gene called DPCD was also likely affected. Furthermore, in a second experiment, mice with deletion of only the catalytic domain of Polλ had a normal phenotype. As a result, the PCD phenotype found in Polλ knockout mice by Kobayashi et al. (2002) is most likely attributable to the loss of DPCD (Zariwala et al., 2004).

## Disorientation of Ependymal Cilia

We know from the preceding description that the aberrant quantity and structure of ependymal cilia can impair CSF. Furthermore, abnormal cilia function in the ependyma will result in the abnormal accumulation of CSF.

Normal ependymal cilia swing in a set polarity direction, moving CSF from the lateral ventricle to the third ventricle, and then through the midbrain aqueduct into the fourth ventricle. When the polarity of ependymal cilia is compromised due to a variety of causes, the equilibrium mode of directional CSF flow will be disrupted, causing CSF to accumulate in the ventricle and prevent it from being evacuated on time.

### Reduction in Ciliary Movement Frequency

Primary cilia are usually ‘9 + 0’ type, whereas motile cilia are ‘9 + 2.’ The most obvious structure to identify the two is CP, which is absolutely necessary for maintaining the function of the motile cilia in the brain (Dawe et al., 2007). The CP apparatus is a complicated structure made up of at least seven distinct protein projections that regulate dynein motion forces and allow the dynein arm to maintain normal ciliary motion (Lee et al., 2008; Brown et al., 2012; McKenzie et al., 2015). As a result, in this review, we concentrate on genes linked to reduction in ciliary movement frequency, which is mainly driven by CP dysfunction.

Hydin is located in the CP of brain and is required for appropriate cilia motility. In Hydin mutant mice, early hydrocephalus symptoms such as enlargement of the lateral ventricle and third ventricle were observed, which is related to aberrant CSF accumulation caused by defective ciliary activity. The ependymal cilia will be unable to bend appropriately due to the lack of a particular projection in Hydin, resulting in a decrease in cilia beating frequency and cilia stagnation (Dawe et al., 2007; Al Omran et al., 2017).

Cilia-and flagella-associated protein 221 (CFAP221), also known as primary ciliary dyskinesia protein 1 (PCDP1), is a CP protein that regulates ependymal ciliary motility and is located to the C1d projection of the CP apparatus. The CFAP221 expression being suppressed will result in a reduction in ependymal ciliary beat frequency (McKenzie and Lee, 2020).

The C1d projection complex is encoded by cilia-and flagella-associated protein 54 (CFAP54), a crucial component of the C1d assembly mechanism. Mutations in CFAP54 cause reduced ependymal ciliary beating frequency (Lee et al., 2008) and loss of the C1d projection (McKenzie and Lee, 2020), leading in CSF flow disruption and accumulation, similar to CFAP221 (McKenzie et al., 2015).

### Planar Cell Polarity Disorder

In ECs, PCP, also known as tissue polarity (Tissir et al., 2010), takes two forms: translational polarity and rotational polarity. The stability of ependymal ciliary motion is dependent on both intracellular and intercellular polarity coordination (Ohata et al., 2014).

The human DAPLE gene determines the direction of CSF flow and regulates the orientation of ependymal cilia (Yamada et al., 2021). DAPLE deficiency impairs horizontal polarization in ECs (Takagishi et al., 2017) as well as the beat of ependymal cilia.

Disheveled (DVLs) are also necessary for preserving the polarity of the EC plane. In ECs, the application of dominant negative disheveled 2 (DVL2) has been found to impair the rotational arrangement of cilia. Furthermore, in adult mice, selectively induced DVLs removal resulted in ependymal cilia with a faulty intracellular rotational configuration. Reduced frequency of ependymal ciliary beat and aberrant CSF accumulation may occur if rotational polarity is disrupted (Ohata et al., 2014).

**FIGURE 2:**
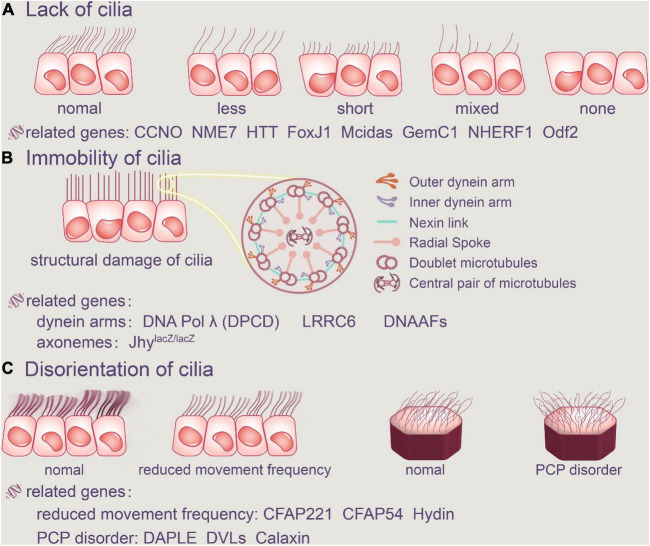
Abnormalities of ependymal cilia. The absence of cilia, cilia immobility, and changes in planar polarity that modify the direction of the cilia’s beat are all examples of ciliary disorders. **(A)** Cilia loss occurs when the number of ependymal cilia decreases, their length decreases, or there are no cilia at all. FoxJ1, Mcidas, GemC1, NHERF1, Odf2, CCNO, NME7, and HTT are all related genes. **(B)** Furthermore, the structure of the original normal cilia is destroyed, as is the aberrant function of the dynein arms and axonemes, resulting in ependymal cilia immobility. DNAAFFs, CCDC151, and DNA Polλ (DPCD) are all related genes. In addition, aberrant cilia function in the ependyma causes inappropriate CSF accumulation. **(C)** Ciliary movement frequency will be reduced by Hydin, CFAP221 and CFAP54, and regular PCP disorder will be harmed. CCNO, Cyclin O; NME7, non-metastatic cell 7; HTT, Huntington protein; LRRC6, leucine-rich repeat-containing protein 6; DNAAFs, dynein axonemal assembly factors; CFAP221, cilia-and flagella-associated protein 221; CFAP54, cilia-and flagella-associated protein 54; DVLs, disheveled.

Calaxin, a Ca2 + binding dynein-related protein that controls cilia motility, binds to ODA directly in a Ca2 + -dependent way. Ependymal cilia in mice lacking the EFCAB1 gene encoding Calaxin had somewhat lower fluid flow rates driven by ependymal cilia than normal ependymal cilia, according to Sasaki et al. (2019). Calaxin deficiency may thereby disrupt the rotational polarity of ependymal cilia, leading to absonant cilia movement (Sasaki et al., 2019; Figure 2, Abnormalities of ependymal cilia).

## Factors Causing Pathophysiology of Motile Cilia in Hydrocephalus

Although it has previously been stated that defects in the structure or function of ependymal cilia can clearly lead to hydrocephalus, here we thoroughly discuss some factors that may be linked to abnormal motile cilia, including alcohol abuse, degenerative neuropathology such as Alzheimer’s disease (AD), PCD, and brain injury (Drielsma et al., 2012; Jiménez et al., 2014; Yamada et al., 2021).

### Alcohol Abuse

Chronic alcohol consumption can have a number of negative effects on the brain, including a 31–71% rise in ventricular size, which is a sign of hydrocephalus (de la Monte, 1988; Zahr et al., 2014). Al Omran et al. (2017) recently found that treating brain slices *in vitro* with 0.25% ethanol reduced the beating frequency of cilia, resulting in a decrease in CSF movement speed. More notably, their research found that ethanol consumption decreased ependymal ciliary pulsation and function in the lateral and third ventricles in rats by reporting three distinct types of ependymal cilia named type-I, type-II, and type-III. These three types were classified based on their frequency and angle of beating, distinct localization in the mouse brain (Al Omran et al., 2017). Ethanol lowers the frequency of ependymal cilia oscillations in rat models, according to findings (Yamada et al., 2021). It can be concluded that ethanol can impair the normal movement of the ependymal cilia in the ventricle, causing hydrocephalus by blocking the circulation of CSF (Saternos and AbouAlaiwi, 2019).

One of the major causes of congenital hydrocephalus is alcohol abuse during pregnancy. Long-term drinking can also have a variety of negative consequences on adults’ brains, including hydrocephalus (Al Omran et al., 2017).

### Alzheimer’s Disease

Normal pressure hydrocephalus (NPH) is a common neurological disease. Hakim’s triad of clinical symptoms includes dementia, gait problem, and urinary incontinence (Johanson et al., 2008; Tan et al., 2021). With aging, the ability of CP epithelial cells to generate CSF decreased. As the rate of CSF formation declines by 50% or more throughout the development of disease, the absorption rate of CSF slows down, resulting in a consequently increase of the concentration of toxic substance. AD is a progressive neurological illness that affects adults over the age of 70 and is characterized by memory disorders, executive dysfunction, and comprehensiveness including dementia, personality changes and behavioral changes (Inaba et al., 2016; Xiao et al., 2021). Because NPH is frequently detected in people with Alzheimer’s disease, the current hypothesis proposes that NPH is the outcome of neurodegenerative alterations in AD patients. The removal of toxic molecules such as Aβ is aided by the circulation of CSF (Zhan et al., 2020).

More ependymal cilia fall off in the ventricle as the aging of the CNS, and the circulation of CSF flow is reduced, resulting in decreased clearance of potentially toxic metabolites, contributing to AD. The poisonous molecule cannot be eliminated quickly enough in AD patients, and it is deposited in the meninges, increasing CSF outflow resistance, inhibiting CSF absorption and accumulating in the ventricle, leading to NPH (Keryer et al., 2011).

Recently, Wilson and Williams believe that iNPH can now be categorized as a reversible dementia since our understanding of the disease has improved. Because the symptoms of iNPH are so similar to those of AD or PD, the majority of patients will be misdiagnosed or go undiscovered. As a result, some scholars have questioned the existence of iNPH (Yang et al., 2021). Even though occurrences of AD-caused hydrocephalus are uncommon, the incidence cannot be ruled out and scholars should delve deeper into this topic.

### Primary Ciliary Dyskinesia

Primary ciliary dyskinesia, formerly known as immobile cilia syndrome, is caused by defects in the structure and function of cilia. Chronic sinusitis, otitis media, infertility, inverted position, and hydrocephalus are among the symptoms that patients may experience. However, the incidence of these related diseases varies greatly among patients with PCD. According to recent reports, the incidence of hydrocephalus in patients with PCD is relatively low when compared to chronic sinusitis, otitis media, male infertility, or inverted position. Hydrocephalus, on the other hand, is a common occurrence in mice with PCD (Lee, 2013). A link between PCD and hydrocephalus has been established by numerous studies (Mulroy et al., 2020).

Due to structural malfunction, the ependymal cilia of PCD patients are unable to beat at a regular frequency, often contributing to the impairment of normal CSF circulation. As a result, accumulated CSF enlarges the ventricle, eventually develops into hydrocephalus.

### Brain Injury

Brain injury is an organic injury to the brain tissue induced by head trauma. When the cranium is struck, the CSF in the ventricle can act as a shock absorber, reducing the risk of brain tissue injury. The continual beating of ependymal cilia contributes to CSF circulation and is the foundation of nutrient exchange and waste elimination, as we already know. Therefore, the sharp reduction of ependymal cilia caused by brain injury may have serious pathological repercussions.

**FIGURE 3:**
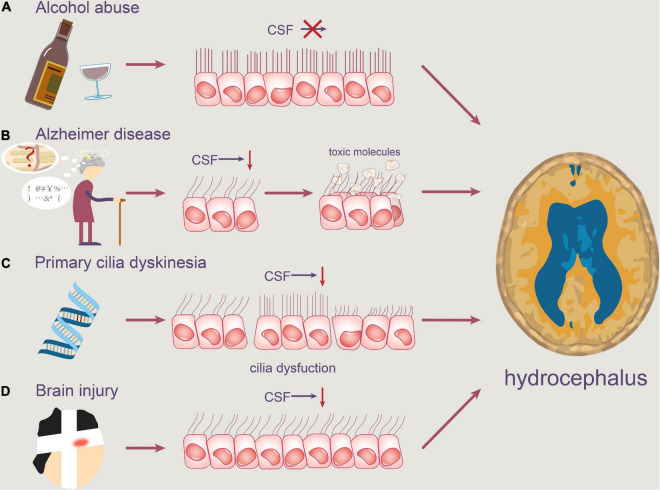
Pathophysiology of motile cilia in hydrocephalus. Abnormal motile cilia can be caused by a variety of reasons. **(A)** Alcohol abuse may reduce the beating frequency of cilia, resulting in slower CSF flow. **(B)** Degenerative neuropathologies, such as Alzheimer’s disease, can obstruct the clearance of toxic molecules like Aβ, causing them to accumulate in the ventricle. **(C)** Furthermore, PCD patients’ ependymal cilia are unable to beat at a regular frequency, which contributes to a disruption in normal CSF circulation. **(D)** Concussion brain injury also contributes to the rapid shedding of ventricular ependymal cilia and the reduction of CSF flow frequency.

In the study of Williamson et al. (2018), collagenase caused intracerebral hemorrhage in the rat model, according to Xiong et al. (2014), concussion brain injury produces rapid shedding of ventricular ependymal cilia and lowers the frequency of CSF flow (Bothwell et al., 2019, 2021). Furthermore, on the surface of the lateral ventricle 1 week after concussion, there were essentially no ependymal cilia (Xiong et al., 2014). Because of the rapid decrease in the number of ependymal cilia induced by brain damage, CSF will accumulate, resulting in hydrocephalus (Figure 3, Pathophysiology of motile cilia in hydrocephalus).

## Treatment of Hydrocephalus by Targeting Ependymal Cilia

The abnormal collection of CSF in the ventricle causes hydrocephalus (Zhan et al., 2020). Ependymal cilia dysfunction can obstruct CSF circulation, causing a discrepancy between CSF production and absorption (Al Omran et al., 2017). The two types of hydrocephalus are communicating and obstructive, with the former being more common. Communicating hydrocephalus may be due to excessive CSF secretion or secondary to reduced CSF absorption.

Medical treatments and surgical management are now used to treat hydrocephalus (Wang et al., 2021). Drug treatment is generally only applicable to patients with mild hydrocephalus and mild hydrocephalus under the age of two. Acetazolamide, as well as dehydration medicines and diuretics including mannitol, hydrochlorothiazide, amino pteridine, and furosemide, are preferred to decrease CSF secretion. A lateral ventricle and third ventricle fistula, a ventricle subarachnoid shunt, repeated puncture of the ventricle through drilling, suboccipital decompression, and other surgical treatments are available (Bhushan et al., 2021).

Despite the fact that surgery is still the most effective treatment for hydrocephalus, it can cause a variety of major problems, including shunt system blockage and infection, as well as a poor prognosis (Johanson et al., 2008; Wu et al., 2020). As a result, it is urgent to find a better treatment.

The moving cilia are recognized to be directly responsible for the unidirectional flow of CSF. The coordinated beating of cilia in a certain direction causes powerful CSF movement, and ependymal cilia dysfunction can be caused by a variety of gene mutations. We summarized the findings from human and experimental animal models from this perspective. We categorized them into several kinds based on many types of ciliary deficiency phenotypes found in various models: reduced ciliary number, shorter cilia, ciliary structural flaws, and ciliary polarity disorder (Lee, 2013; Kumar et al., 2021). Different phenotypes share a common characteristic: they disrupt the normal movement of ependymal cilia, causing further harm to the CSF circulation and resulting in hydrocephalus. We hope that by focusing on these harmful genes, we may be able to develop targeted therapies for specific mutation locations or action pathways, to further correct ependymal cilia defects and alleviate hydrocephalus. Table 1 lists the genes that have been identified as playing a role or that may play a role in ependymal cilia, as well as their relationship with ependymal ciliary dysfunction (Table 1, A comprehensive list of ependymal cilia dysfunction-related genes).

**TABLE 1:** A comprehensive list of ependymal cilia dysfunction-related genes.

Gene	Protein localization/Function	Structural defect in ependymal cilia	Typical functional defect in ependymal cilia	References
FoxJ1	Transcription factor	Inability of centrioles to be transported to the surface of ECs	Lack of ependymal cilia	Jacquet et al., 2009; Lewis and Stracker, 2021
Mcidas	Transcription factor	Inability to extend centrioles	Lack of ependymal cilia	Lewis and Stracker, 2021; Omiya et al., 2021
GemC1	Transcription factor	Inability to extend centrioles	Lack of ependymal cilia	Lewis and Stracker, 2021; Omiya et al., 2021
NHERF1	Apical cytoplasm	Inability to establishes protein complexes at the apical PM	Lack of ependymal cilia	Georgescu et al., 2015
Odf2	BF in the polarized organization	Inability of formation of BF	Lack of ependymal cilia	Kunimoto et al., 2012
CCNO	Apical cytoplasm	The reduction of the number of centrioles	Lack of ependymal cilia	Wallmeier et al., 2014; Funk et al., 2015; Núnez-Ollé et al., 2017
NME7	MT organizing center	The reduction of the number of centrioles	Lack of ependymal cilia	Liu et al., 2014; Šedová et al., 2021
Jhy	Unknown	Altered structure of axonemes	Immotile ependymal cilia	Appelbe et al., 2013; Muniz-Talavera and Schmidt, 2017
DNAAF1/LRRC50/ODA7	Cytoplasmic, DA assembling	ODA + IDA defect	Immotile ependymal cilia	Duquesnoy et al., 2009; Loges et al., 2009; Ha et al., 2016
DNAAF2/PF13/KTU	Cytoplasmic, DA assembling	ODA + IDA defect	Immotile ependymal cilia	Omran et al., 2008; Ha et al., 2016
DNAAF3/PF22	Cytoplasmic, DA assembling	ODA + IDA defect	Immotile ependymal cilia	Mitchison et al., 2012; Ha et al., 2016
DNAAF4/DYX1C1	Cytoplasmic, DA assembling	ODA + IDA defect	Immotile ependymal cilia	Chandrasekar et al., 2013; Ha et al., 2016; Cho et al., 2018
LRRC6	Cytoplasmic, DA assembling	ODA + IDA defect	Immotile ependymal cilia	Kott et al., 2012; Inaba et al., 2013, 2016
CCDC151	ODA targeting and docking	ODA defect	Immotile ependymal cilia	Alsaadi et al., 2014; Hjeij et al., 2014; Chiani et al., 2019
DNAI1	ODA	ODA defect	Immotile ependymal cilia	Horváth et al., 2005; Zariwala et al., 2006; Failly et al., 2008; Ziêtkiewicz et al., 2010; Mazor et al., 2011; Wildung et al., 2019; Bhushan et al., 2021
DNAI2	ODA	ODA defect	Immotile ependymal cilia	Rocca et al., 2008, 2020
ZMYND10	Cytoplasmic, DA assembling	ODA + IDA defect	Immotile ependymal cilia	Rocca et al., 2008; Moore et al., 2013; Zariwala et al., 2013; Cho et al., 2018
CCDC39	Nexin-dynein regulatory complexes (N-DRCs)	MT disorganization and IDA defect	Short and immotile ependymal cilia	Merveille et al., 2011; Abdelhamed et al., 2018; Emmert et al., 2019
DNA Polλ	DNA repair polymerase	IDA defect	Immotile ependymal cilia	Kobayashi et al., 2002
RSPH9	Radial spokes (RSs)	MT disorganization (CP-RS defect)	Lower beating amplitude and disorientation of ependymal cilia	Castleman et al., 2009; Ziêtkiewicz et al., 2012; Kott et al., 2013; Zou et al., 2020
Hydin	CP	Reduction of ciliary movement frequency	Disorientation of ependymal cilia	Dawe et al., 2007; Al Omran et al., 2017
CFAP221/PCDP1	CP	Reduction of ciliary movement frequency	Disorientation of ependymal cilia	Lee et al., 2008; DiPetrillo and Smith, 2010; McKenzie et al., 2018; McKenzie and Lee, 2020
CFAP54	CP	Reduction of ciliary movement frequency	Disorientation of ependymal cilia	Lee et al., 2008; McKenzie et al., 2015, 2018; McKenzie and Lee, 2020
DAPLE	Anterior side of the apical membrane	PCP disorder	Disorientation of ependymal cilia	Takagishi et al., 2017
DVLs	Unknown	PCP disorder	Disorientation of ependymal cilia	Ohata et al., 2014; Yamada et al., 2021
EFCAB1	Unknown	PCP disorder	Disorientation of ependymal cilia	Sasaki et al., 2019

## Conclusion

We outlined the primary physiological activities of ependymal cilia in this review, which include aiding CSF circulation and regulating neuroblast migration. Recent results related to ciliogenesis dysfunctions, on the other hand, have provided new insights into how it causes disorders like AD and HD.

Furthermore, the abnormalities of ependymal cilia are linked to the occurrence of hydrocephalus, whose mechanism is currently understood as a disruption of normal CSF flow, which leads to aberrant accumulation in the brain. The relationship between phenotypes and genotypes of ependymal cilia should be clarified from a broad perspective. As a result, we’ve identified the linked causative genes associated with hydrocephalus in Table 1 for future research. The discovery of these genes will not only aid in the genetic knowledge of cilia-related hydrocephalus, but it may also provide a unique latent point for future hydrocephalus diagnostic and therapy strategies.

In conclusion, we think that this review will be useful in determining the precise mechanism of cilia-caused hydrocephalus. Despite the fact that our current understanding is still superficial, it is expected that more basic and clinical research will emerge in the future, which will assist people all around the world.

## Author Contributions

WYJ and ZT collected the related manuscript. WYJ, ZT, YBC, CSW, JBL, LT, and CWT drafted and revised the manuscript. GX participated in the design of the review and helped to draft and revise the manuscript. All authors read and approved the final manuscript.

## Conflict of Interest

The authors declare that the research was conducted in the absence of any commercial or financial relationships that could be construed as a potential conflict of interest.

## Publisher’s Note

All claims expressed in this article are solely those of the authors and do not necessarily represent those of their affiliated organizations, or those of the publisher, the editors and the reviewers. Any product that may be evaluated in this article, or claim that may be made by its manufacturer, is not guaranteed or endorsed by the publisher.

## Abbreviations

CSF: cerebrospinal fluid
ECs: ependymal cells
PCD: primary ciliary dyskinesia
CNS: central nervous system
BBs: basal bodies
BF: basal feet
PM: plasma membrane
ODF: outer dense fiber
CCNO: Cyclin O
NME7: non-metastatic cell 7
γ TuRC: γ-tubulin ring complex
MT: microtubule
HTT: Huntington protein
HAP1: Huntington-associated protein 1
PCM1: perinuclear matter 1 protein
HD: Huntington disease
CP: central pair
PMTs: peripheral microtubules
ODA: outer dynein arm
IDA: inner dynein arm
DNAAFs: dynein axonemal assembly factors
ODA7: outer dynein arm 7
LRRC50: leucine-rich repeat-containing protein 50
KTU: kintoun
LRRC6: leucine-rich repeat-containing protein 6
CCDC151: coiled-coil domain-containing protein 151
CFAP221: cilia-and flagella-associated protein 221
PCDP1: primary ciliary dyskinesia protein 1
CFAP54: cilia-and flagella-associated protein 54
PCP: planar cell polarity
DVLs: disheveled)
DVL2: disheveled 2
AD: Alzheimer’s disease
NPH: normal pressure hydrocephalus
N-DRCs: nexin-dynein regulatory complexes
RS: radial spoke.
